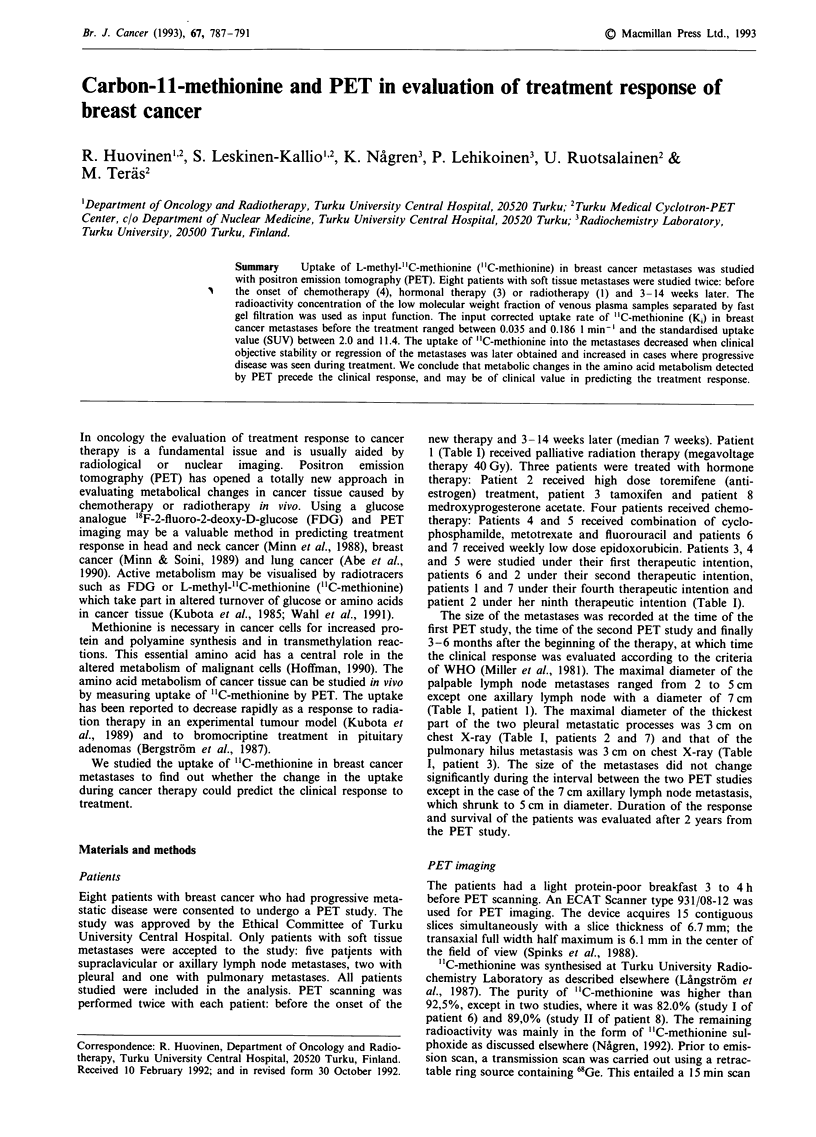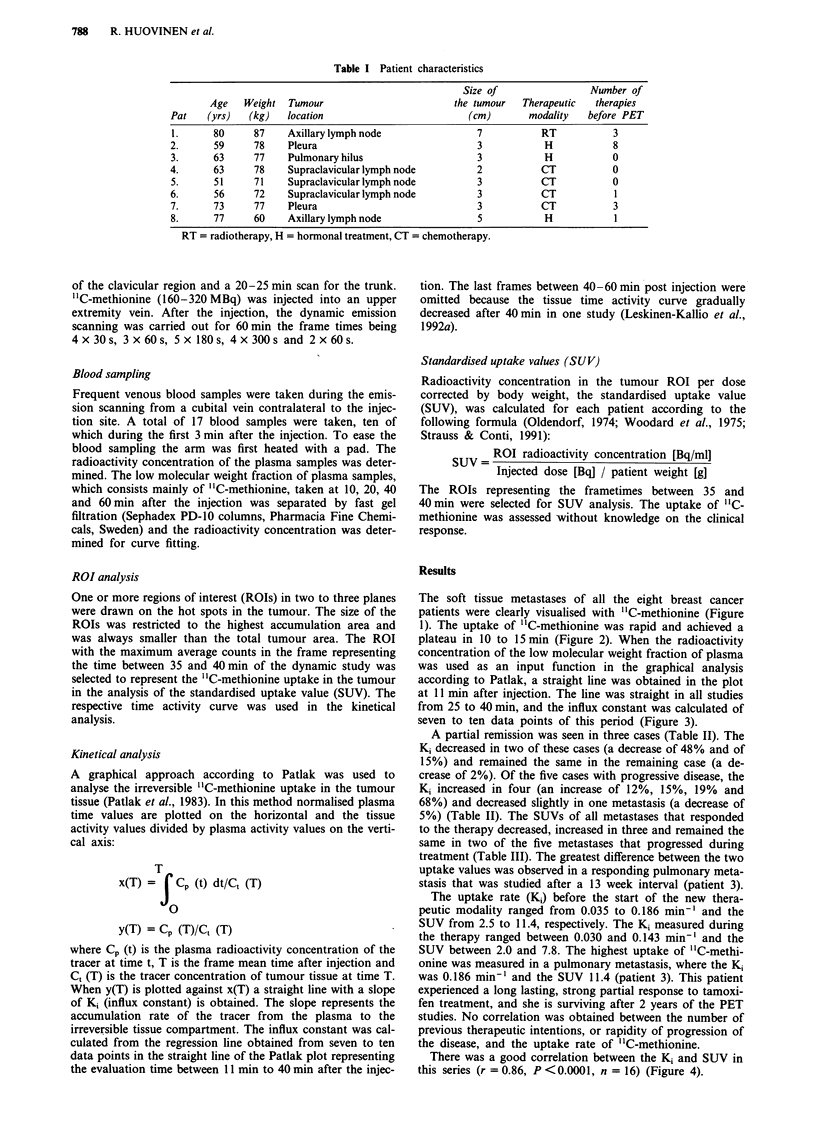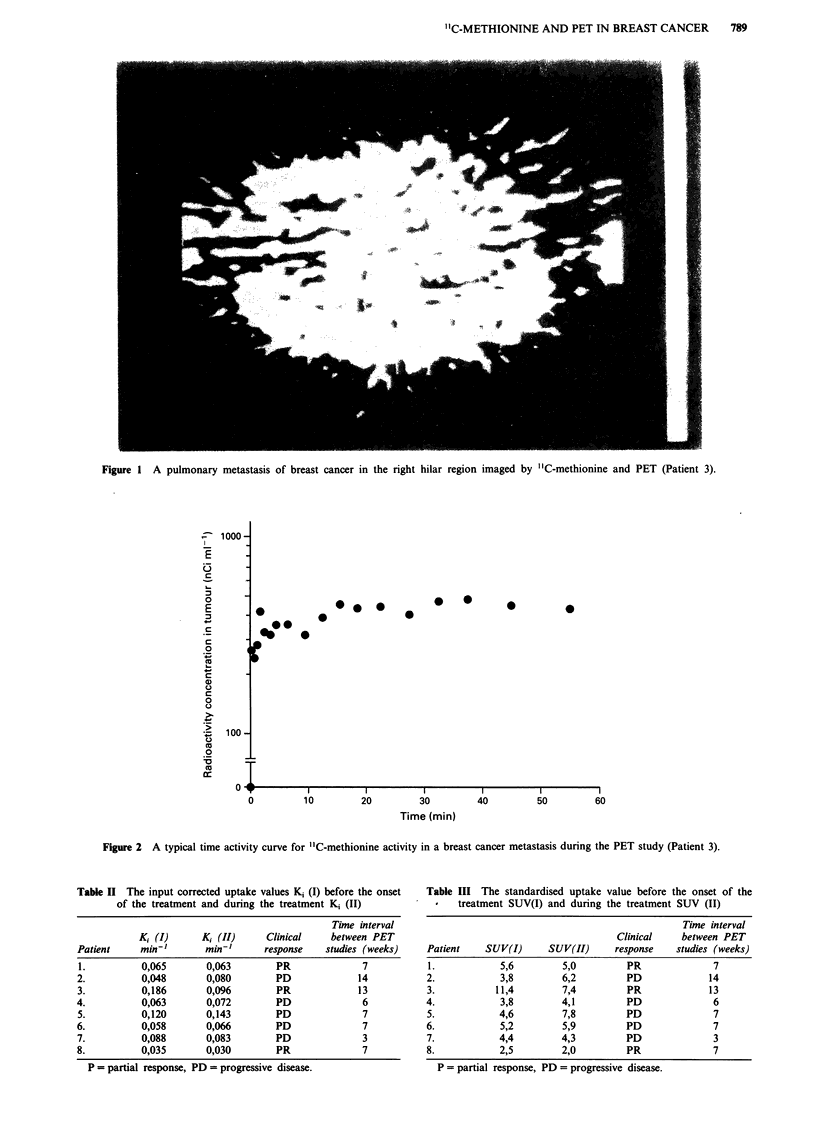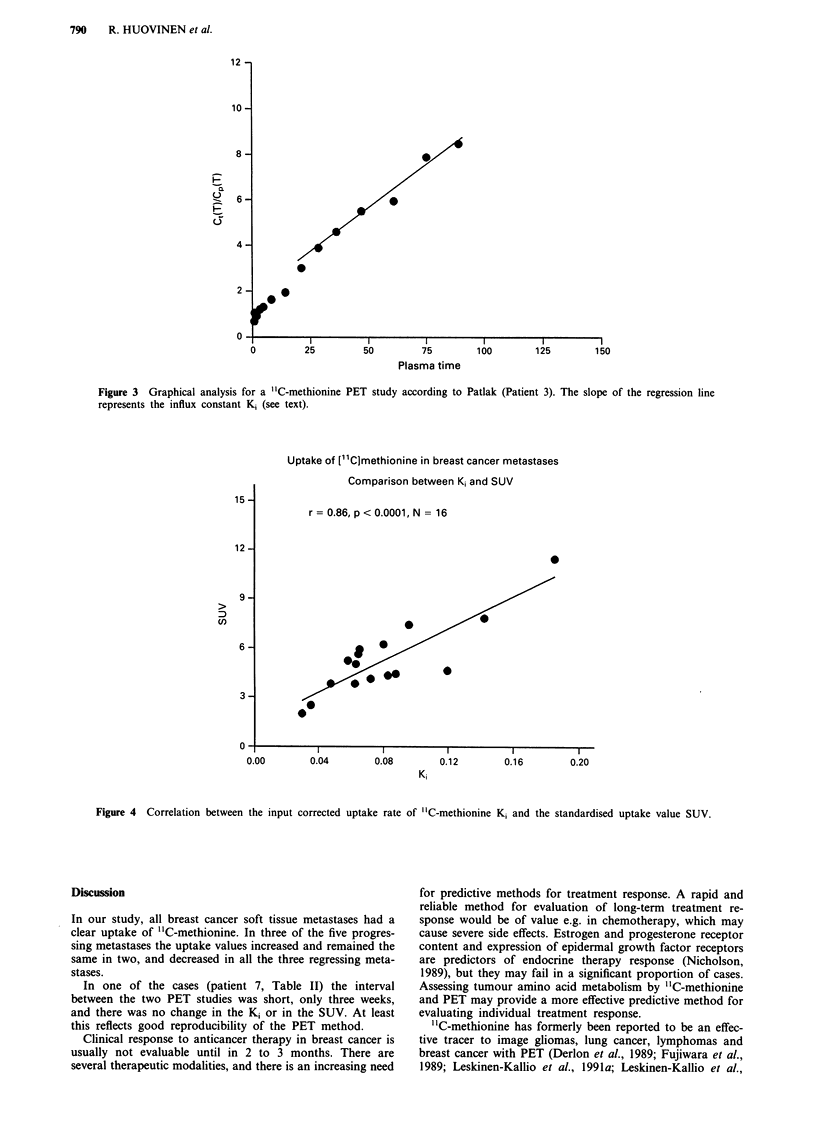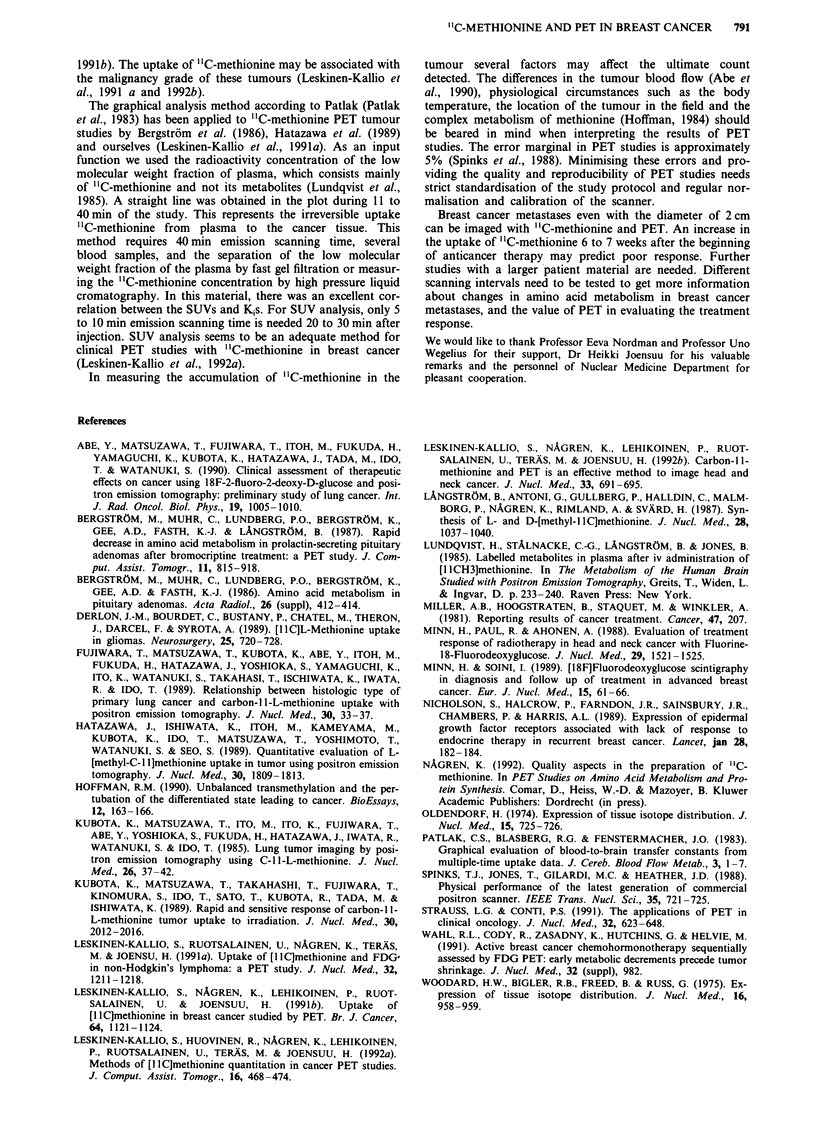# Carbon-11-methionine and PET in evaluation of treatment response of breast cancer.

**DOI:** 10.1038/bjc.1993.143

**Published:** 1993-04

**Authors:** R. Huovinen, S. Leskinen-Kallio, K. Någren, P. Lehikoinen, U. Ruotsalainen, M. Teräs

**Affiliations:** Department of Oncology and Radiotherapy, Turku University Central Hospital, Finland.

## Abstract

**Images:**


 © Macmillan Press Ltd., 1993

### Carbon-11-methionine and PET in evaluation of treatment response of breast cancer

R. Huovinen[1,2], S. Leskinen-Kallio[1,2], K. Någren[3], P. Lehikoinen[3], U. Ruotsalainen[2] & M. Teräs[2]

[1]*Department of Oncology and Radiotherapy, Turku University Central Hospital, 20520 Turku;* [2]*Turku Medical Cyclotron-PET Center, c/o Department of Nuclear Medicine, Turku University Central Hospital, 20520 Turku;* [3]*Radiochemistry Laboratory, Turku University, 20500 Turku, Finland.*

**Summary** Uptake of L-methyl-$^{11}$C-methionine ($^{11}$C-methionine) in breast cancer metastases was studied with positron emission tomography (PET). Eight patients with soft tissue metastases were studied twice: before the onset of chemotherapy (4), hormonal therapy (3) or radiotherapy (1) and 3-14 weeks later. The radioactivity concentration of the low molecular weight fraction of venous plasma samples separated by fast gel filtration was used as input function. The input corrected uptake rate of $^{11}$C-methionine ($K_i$) in breast cancer metastases before the treatment ranged between 0.035 and 0.186 l min$^{-1}$ and the standardised uptake value (SUV) between 2.0 and 11.4. The uptake of $^{11}$C-methionine into the metastases decreased when clinical objective stability or regression of the metastases was later obtained and increased in cases where progressive disease was seen during treatment. We conclude that metabolic changes in the amino acid metabolism detected by PET precede the clinical response, and may be of clinical value in predicting the treatment response.

In oncology the evaluation of treatment response to cancer therapy is a fundamental issue and is usually aided by radiological or nuclear imaging. Positron emission tomography (PET) has opened a totally new approach in evaluating metabolical changes in cancer tissue caused by chemotherapy or radiotherapy *in vivo*. Using a glucose analogue $^{18}$F-2-fluoro-2-deoxy-D-glucose (FDG) and PET imaging may be a valuable method in predicting treatment response in head and neck cancer (Minn *et al.*, 1988), breast cancer (Minn & Soini, 1989) and lung cancer (Abe *et al.*, 1990). Active metabolism may be visualised by radiotracers such as FDG or L-methyl-$^{11}$C-methionine ($^{11}$C-methionine) which take part in altered turnover of glucose or amino acids in cancer tissue (Kubota *et al.*, 1985; Wahl *et al.*, 1991).

Methionine is necessary in cancer cells for increased protein and polyamine synthesis and in transmethylation reactions. This essential amino acid has a central role in the altered metabolism of malignant cells (Hoffman, 1990). The amino acid metabolism of cancer tissue can be studied *in vivo* by measuring uptake of $^{11}$C-methionine by PET. The uptake has been reported to decrease rapidly as a response to radiation therapy in an experimental tumour model (Kubota *et al.*, 1989) and to bromocriptine treatment in pituitary adenomas (Bergström *et al.*, 1987).

We studied the uptake of $^{11}$C-methionine in breast cancer metastases to find out whether the change in the uptake during cancer therapy could predict the clinical response to treatment.

### Materials and methods

### Patients

Eight patients with breast cancer who had progressive metastatic disease were consented to undergo a PET study. The study was approved by the Ethical Committee of Turku University Central Hospital. Only patients with soft tissue metastases were accepted to the study: five patients with supraclavicular or axillary lymph node metastases, two with pleural and one with pulmonary metastases. All patients studied were included in the analysis. PET scanning was performed twice with each patient: before the onset of the new therapy and 3-14 weeks later (median 7 weeks). Patient 1 (Table I) received palliative radiation therapy (megavoltage therapy 40 Gy). Three patients were treated with hormone therapy: Patient 2 received high dose toremifene (anti-estrogen) treatment, patient 3 tamoxifen and patient 8 medroxyprogesterone acetate. Four patients received chemotherapy: Patients 4 and 5 received combination of cyclophosphamilde, metotrexate and fluorouracil and patients 6 and 7 received weekly low dose epidoxorubicin. Patients 3, 4 and 5 were studied under their first therapeutic intention, patients 6 and 2 under their second therapeutic intention, patients 1 and 7 under their fourth therapeutic intention and patient 2 under her ninth therapeutic intention (Table I).

The size of the metastases was recorded at the time of the first PET study, the time of the second PET study and finally 3-6 months after the beginning of the therapy, at which time the clinical response was evaluated according to the criteria of WHO (Miller *et al.*, 1981). The maximal diameter of the palpable lymph node metastases ranged from 2 to 5 cm except one axillary lymph node with a diameter of 7 cm (Table I, patient 1). The maximal diameter of the thickest part of the two pleural metastatic processes was 3 cm on chest X-ray (Table I, patients 2 and 7) and that of the pulmonary hilus metastasis was 3 cm on chest X-ray (Table I, patient 3). The size of the metastases did not change significantly during the interval between the two PET studies except in the case of the 7 cm axillary lymph node metastasis, which shrunk to 5 cm in diameter. Duration of the response and survival of the patients was evaluated after 2 years from the PET study.

### PET imaging

The patients had a light protein-poor breakfast 3 to 4 h before PET scanning. An ECAT Scanner type 931/08-12 was used for PET imaging. The device acquires 15 contiguous slices simultaneously with a slice thickness of 6.7 mm; the transaxial full width half maximum is 6.1 mm in the center of the field of view (Spinks *et al.*, 1988).

$^{11}$C-methionine was synthesised at Turku University Radiochemistry Laboratory as described elsewhere (Långström *et al.*, 1987). The purity of $^{11}$C-methionine was higher than 92,5%, except in two studies, where it was 82.0% (study I of patient 6) and 89,0% (study II of patient 8). The remaining radioactivity was mainly in the form of $^{11}$C-methionine sulphoxide as discussed elsewhere (Någren, 1992). Prior to emission scan, a transmission scan was carried out using a retractable ring source containing $^{68}$Ge. This entailed a 15 min scan

Correspondence: R. Huovinen, Department of Oncology and Radiotherapy, Turku University Central Hospital, 20520 Turku, Finland.
Received 10 February 1992; and in revised form 30 October 1992.

**Table I** Patient characteristics

| Pat | Age (yrs) | Weight (kg) | Tumour location | Size of the tumour (cm) | Therapeutic modality | Number of therapies before PET |
|---|---|---|---|---|---|---|
| 1. | 80 | 87 | Axillary lymph node | 7 | RT | 3 |
| 2. | 59 | 78 | Pleura | 3 | H | 8 |
| 3. | 63 | 77 | Pulmonary hilus | 3 | H | 0 |
| 4. | 63 | 78 | Supraclavicular lymph node | 2 | CT | 0 |
| 5. | 51 | 71 | Supraclavicular lymph node | 3 | CT | 0 |
| 6. | 56 | 72 | Supraclavicular lymph node | 3 | CT | 1 |
| 7. | 73 | 77 | Pleura | 3 | CT | 3 |
| 8. | 77 | 60 | Axillary lymph node | 5 | H | 1 |

RT = radiotherapy, H = hormonal treatment, CT = chemotherapy.

of the clavicular region and a 20–25 min scan for the trunk. $^{11}$C-methionine (160–320 MBq) was injected into an upper extremity vein. After the injection, the dynamic emission scanning was carried out for 60 min the frame times being 4 × 30 s, 3 × 60 s, 5 × 180 s, 4 × 300 s and 2 × 60 s.

*Blood sampling*

Frequent venous blood samples were taken during the emission scanning from a cubital vein contralateral to the injection site. A total of 17 blood samples were taken, ten of which during the first 3 min after the injection. To ease the blood sampling the arm was first heated with a pad. The radioactivity concentration of the plasma samples was determined. The low molecular weight fraction of plasma samples, which consists mainly of $^{11}$C-methionine, taken at 10, 20, 40 and 60 min after the injection was separated by fast gel filtration (Sephadex PD-10 columns, Pharmacia Fine Chemicals, Sweden) and the radioactivity concentration was determined for curve fitting.

*ROI analysis*

One or more regions of interest (ROIs) in two to three planes were drawn on the hot spots in the tumour. The size of the ROIs was restricted to the highest accumulation area and was always smaller than the total tumour area. The ROI with the maximum average counts in the frame representing the time between 35 and 40 min of the dynamic study was selected to represent the $^{11}$C-methionine uptake in the tumour in the analysis of the standardised uptake value (SUV). The respective time activity curve was used in the kinetical analysis.

*Kinetical analysis*

A graphical approach according to Patlak was used to analyse the irreversible $^{11}$C-methionine uptake in the tumour tissue (Patlak *et al.*, 1983). In this method normalised plasma time values are plotted on the horizontal and the tissue activity values divided by plasma activity values on the vertical axis:

$$x(T) = \int_{O}^{T} C_p\ (t)\ dt / C_t\ (T)$$

$$y(T) = C_p\ (T)/C_t\ (T)$$

where $C_p$ (t) is the plasma radioactivity concentration of the tracer at time t, T is the frame mean time after injection and $C_t$ (T) is the tracer concentration of tumour tissue at time T. When y(T) is plotted against x(T) a straight line with a slope of $K_i$ (influx constant) is obtained. The slope represents the accumulation rate of the tracer from the plasma to the irreversible tissue compartment. The influx constant was calculated from the regression line obtained from seven to ten data points in the straight line of the Patlak plot representing the evaluation time between 11 min to 40 min after the injection. The last frames between 40–60 min post injection were omitted because the tissue time activity curve gradually decreased after 40 min in one study (Leskinen-Kallio *et al.*, 1992*a*).

*Standardised uptake values (SUV)*

Radioactivity concentration in the tumour ROI per dose corrected by body weight, the standardised uptake value (SUV), was calculated for each patient according to the following formula (Oldendorf, 1974; Woodard *et al.*, 1975; Strauss & Conti, 1991):

$$SUV = \frac{\text{ROI radioactivity concentration [Bq/ml]}}{\text{Injected dose [Bq] / patient weight [g]}}$$

The ROIs representing the frametimes between 35 and 40 min were selected for SUV analysis. The uptake of $^{11}$C-methionine was assessed without knowledge on the clinical response.

### Results

The soft tissue metastases of all the eight breast cancer patients were clearly visualised with $^{11}$C-methionine (Figure 1). The uptake of $^{11}$C-methionine was rapid and achieved a plateau in 10 to 15 min (Figure 2). When the radioactivity concentration of the low molecular weight fraction of plasma was used as an input function in the graphical analysis according to Patlak, a straight line was obtained in the plot at 11 min after injection. The line was straight in all studies from 25 to 40 min, and the influx constant was calculated of seven to ten data points of this period (Figure 3).

A partial remission was seen in three cases (Table II). The $K_i$ decreased in two of these cases (a decrease of 48% and of 15%) and remained the same in the remaining case (a decrease of 2%). Of the five cases with progressive disease, the $K_i$ increased in four (an increase of 12%, 15%, 19% and 68%) and decreased slightly in one metastasis (a decrease of 5%) (Table II). The SUVs of all metastases that responded to the therapy decreased, increased in three and remained the same in two of the five metastases that progressed during treatment (Table III). The greatest difference between the two uptake values was observed in a responding pulmonary metastasis that was studied after a 13 week interval (patient 3).

The uptake rate ($K_i$) before the start of the new therapeutic modality ranged from 0.035 to 0.186 min$^{-1}$ and the SUV from 2.5 to 11.4, respectively. The $K_i$ measured during the therapy ranged between 0.030 and 0.143 min$^{-1}$ and the SUV between 2.0 and 7.8. The highest uptake of $^{11}$C-methionine was measured in a pulmonary metastasis, where the $K_i$ was 0.186 min$^{-1}$ and the SUV 11.4 (patient 3). This patient experienced a long lasting, strong partial response to tamoxifen treatment, and she is surviving after 2 years of the PET studies. No correlation was obtained between the number of previous therapeutic intentions, or rapidity of progression of the disease, and the uptake rate of $^{11}$C-methionine.

There was a good correlation between the $K_i$ and SUV in this series ($r = 0.86$, $P < 0.0001$, $n = 16$) (Figure 4).

**Figure 1** A pulmonary metastasis of breast cancer in the right hilar region imaged by $^{11}$C-methionine and PET (Patient 3).

**Figure 2** A typical time activity curve for $^{11}$C-methionine activity in a breast cancer metastasis during the PET study (Patient 3).

**Table II** The input corrected uptake values $K_i$ (I) before the onset of the treatment and during the treatment $K_i$ (II)

| *Patient* | *$K_i$ (I) min$^{-1}$* | *$K_i$ (II) min$^{-1}$* | *Clinical response* | *Time interval between PET studies (weeks)* |
|---|---|---|---|---|
| 1. | 0,065 | 0,063 | PR | 7 |
| 2. | 0,048 | 0,080 | PD | 14 |
| 3. | 0,186 | 0,096 | PR | 13 |
| 4. | 0,063 | 0,072 | PD | 6 |
| 5. | 0,120 | 0,143 | PD | 7 |
| 6. | 0,058 | 0,066 | PD | 7 |
| 7. | 0,088 | 0,083 | PD | 3 |
| 8. | 0,035 | 0,030 | PR | 7 |

P = partial response, PD = progressive disease.

**Table III** The standardised uptake value before the onset of the treatment SUV(I) and during the treatment SUV (II)

| *Patient* | *SUV(I)* | *SUV(II)* | *Clinical response* | *Time interval between PET studies (weeks)* |
|---|---|---|---|---|
| 1. | 5,6 | 5,0 | PR | 7 |
| 2. | 3,8 | 6,2 | PD | 14 |
| 3. | 11,4 | 7,4 | PR | 13 |
| 4. | 3,8 | 4,1 | PD | 6 |
| 5. | 4,6 | 7,8 | PD | 7 |
| 6. | 5,2 | 5,9 | PD | 7 |
| 7. | 4,4 | 4,3 | PD | 3 |
| 8. | 2,5 | 2,0 | PR | 7 |

P = partial response, PD = progressive disease.

**Figure 3** Graphical analysis for a [11]C-methionine PET study according to Patlak (Patient 3). The slope of the regression line represents the influx constant $K_i$ (see text).

**Figure 4** Correlation between the input corrected uptake rate of [11]C-methionine $K_i$ and the standardised uptake value SUV.

### Discussion

In our study, all breast cancer soft tissue metastases had a clear uptake of [11]C-methionine. In three of the five progressing metastases the uptake values increased and remained the same in two, and decreased in all the three regressing metastases.

In one of the cases (patient 7, Table II) the interval between the two PET studies was short, only three weeks, and there was no change in the $K_i$ or in the SUV. At least this reflects good reproducibility of the PET method.

Clinical response to anticancer therapy in breast cancer is usually not evaluable until in 2 to 3 months. There are several therapeutic modalities, and there is an increasing need for predictive methods for treatment response. A rapid and reliable method for evaluation of long-term treatment response would be of value e.g. in chemotherapy, which may cause severe side effects. Estrogen and progesterone receptor content and expression of epidermal growth factor receptors are predictors of endocrine therapy response (Nicholson, 1989), but they may fail in a significant proportion of cases. Assessing tumour amino acid metabolism by [11]C-methionine and PET may provide a more effective predictive method for evaluating individual treatment response.

[11]C-methionine has formerly been reported to be an effective tracer to image gliomas, lung cancer, lymphomas and breast cancer with PET (Derlon et al., 1989; Fujiwara et al., 1989; Leskinen-Kallio et al., 1991a; Leskinen-Kallio et al.,

1991*b*). The uptake of $^{11}$C-methionine may be associated with the malignancy grade of these tumours (Leskinen-Kallio *et al.*, 1991 *a* and 1992*b*).

The graphical analysis method according to Patlak (Patlak *et al.*, 1983) has been applied to $^{11}$C-methionine PET tumour studies by Bergström *et al.* (1986), Hatazawa *et al.* (1989) and ourselves (Leskinen-Kallio *et al.*, 1991*a*). As an input function we used the radioactivity concentration of the low molecular weight fraction of plasma, which consists mainly of $^{11}$C-methionine and not its metabolites (Lundqvist *et al.*, 1985). A straight line was obtained in the plot during 11 to 40 min of the study. This represents the irreversible uptake $^{11}$C-methionine from plasma to the cancer tissue. This method requires 40 min emission scanning time, several blood samples, and the separation of the low molecular weight fraction of the plasma by fast gel filtration or measuring the $^{11}$C-methionine concentration by high pressure liquid cromatography. In this material, there was an excellent correlation between the SUVs and $K_i$s. For SUV analysis, only 5 to 10 min emission scanning time is needed 20 to 30 min after injection. SUV analysis seems to be an adequate method for clinical PET studies with $^{11}$C-methionine in breast cancer (Leskinen-Kallio *et al.*, 1992*a*).

In measuring the accumulation of $^{11}$C-methionine in the tumour several factors may affect the ultimate count detected. The differences in the tumour blood flow (Abe *et al.*, 1990), physiological circumstances such as the body temperature, the location of the tumour in the field and the complex metabolism of methionine (Hoffman, 1984) should be beared in mind when interpreting the results of PET studies. The error marginal in PET studies is approximately 5% (Spinks *et al.*, 1988). Minimising these errors and providing the quality and reproducibility of PET studies needs strict standardisation of the study protocol and regular normalisation and calibration of the scanner.

Breast cancer metastases even with the diameter of 2 cm can be imaged with $^{11}$C-methionine and PET. An increase in the uptake of $^{11}$C-methionine 6 to 7 weeks after the beginning of anticancer therapy may predict poor response. Further studies with a larger patient material are needed. Different scanning intervals need to be tested to get more information about changes in amino acid metabolism in breast cancer metastases, and the value of PET in evaluating the treatment response.

We would like to thank Professor Eeva Nordman and Professor Uno Wegelius for their support, Dr Heikki Joensuu for his valuable remarks and the personnel of Nuclear Medicine Department for pleasant cooperation.

### References

ABE, Y., MATSUZAWA, T., FUJIWARA, T., ITOH, M., FUKUDA, H., YAMAGUCHI, K., KUBOTA, K., HATAZAWA, J., TADA, M., IDO, T. & WATANUKI, S. (1990). Clinical assessment of therapeutic effects on cancer using 18F-2-fluoro-2-deoxy-D-glucose and positron emission tomography: preliminary study of lung cancer. *Int. J. Rad. Oncol. Biol. Phys.*, **19**, 1005–1010.

BERGSTRÖM, M., MUHR, C., LUNDBERG, P.O., BERGSTRÖM, K., GEE, A.D., FASTH, K.-J. & LÅNGSTRÖM, B. (1987). Rapid decrease in amino acid metabolism in prolactin-secreting pituitary adenomas after bromocriptine treatment: a PET study. *J. Comput. Assist. Tomogr.*, **11**, 815–918.

BERGSTRÖM, M., MUHR, C., LUNDBERG, P.O., BERGSTRÖM, K., GEE, A.D. & FASTH, K.-J. (1986). Amino acid metabolism in pituitary adenomas. *Acta Radiol.*, **26** (suppl), 412–414.

DERLON, J.-M., BOURDET, C., BUSTANY, P., CHATEL, M., THERON, J., DARCEL, F. & SYROTA, A. (1989). [11C]L-Methionine uptake in gliomas. *Neurosurgery*, **25**, 720–728.

FUJIWARA, T., MATSUZAWA, T., KUBOTA, K., ABE, Y., ITOH, M., FUKUDA, H., HATAZAWA, J., YOSHIOKA, S., YAMAGUCHI, K., ITO, K., WATANUKI, S., TAKAHASI, T., ISCHIWATA, K., IWATA, R. & IDO, T. (1989). Relationship between histologic type of primary lung cancer and carbon-11-L-methionine uptake with positron emission tomography. *J. Nucl. Med.*, **30**, 33–37.

HATAZAWA, J., ISHIWATA, K., ITOH, M., KAMEYAMA, M., KUBOTA, K., IDO, T., MATSUZAWA, T., YOSHIMOTO, T., WATANUKI, S. & SEO, S. (1989). Quantitative evaluation of L-[methyl-C-11]methionine uptake in tumor using positron emission tomography. *J. Nucl. Med.*, **30**, 1809–1813.

HOFFMAN, R.M. (1990). Unbalanced transmethylation and the pertubation of the differentiated state leading to cancer. *BioEssays*, **12**, 163–166.

KUBOTA, K., MATSUZAWA, T., ITO, M., ITO, K., FUJIWARA, T., ABE, Y., YOSHIOKA, S., FUKUDA, H., HATAZAWA, J., IWATA, R., WATANUKI, S. & IDO, T. (1985). Lung tumor imaging by positron emission tomography using C-11-L-methionine. *J. Nucl. Med.*, **26**, 37–42.

KUBOTA, K., MATSUZAWA, T., TAKAHASHI, T., FUJIWARA, T., KINOMURA, S., IDO, T., SATO, T., KUBOTA, R., TADA, M. & ISHIWATA, K. (1989). Rapid and sensitive response of carbon-11-L-methionine tumor uptake to irradiation. *J. Nucl. Med.*, **30**, 2012–2016.

LESKINEN-KALLIO, S., RUOTSALAINEN, U., NÅGREN, K., TERÄS, M. & JOENSU, H. (1991*a*). Uptake of [11C]methionine and FDG in non-Hodgkin's lymphoma: a PET study. *J. Nucl. Med.*, **32**, 1211–1218.

LESKINEN-KALLIO, S., NÅGREN, K., LEHIKOINEN, P., RUOTSALAINEN, U. & JOENSUU, H. (1991*b*). Uptake of [11C]methionine in breast cancer studied by PET. *Br. J. Cancer*, **64**, 1121–1124.

LESKINEN-KALLIO, S., HUOVINEN, R., NÅGREN, K., LEHIKOINEN, P., RUOTSALAINEN, U., TERÄS, M. & JOENSUU, H. (1992*a*). Methods of [11C]methionine quantitation in cancer PET studies. *J. Comput. Assist. Tomogr.*, **16**, 468–474.

LESKINEN-KALLIO, S., NÅGREN, K., LEHIKOINEN, P., RUOTSALAINEN, U., TERÄS, M. & JOENSUU, H. (1992*b*). Carbon-11-methionine and PET is an effective method to image head and neck cancer. *J. Nucl. Med.*, **33**, 691–695.

LÅNGSTRÖM, B., ANTONI, G., GULLBERG, P., HALLDIN, C., MALMBORG, P., NÅGREN, K., RIMLAND, A. & SVÄRD, H. (1987). Synthesis of L- and D-[methyl-11C]methionine. *J. Nucl. Med.*, **28**, 1037–1040.

LUNDQVIST, H., STÅLNACKE, C.-G., LÅNGSTRÖM, B. & JONES, B. (1985). Labelled metabolites in plasma after iv administration of [11CH3]methionine. In *The Metabolism of the Human Brain Studied with Positron Emission Tomography*, Greits, T., Widen, L. & Ingvar, D. p. 233–240. Raven Press: New York.

MILLER, A.B., HOOGSTRATEN, B., STAQUET, M. & WINKLER, A. (1981). Reporting results of cancer treatment. *Cancer*, **47**, 207.

MINN, H., PAUL, R. & AHONEN, A. (1988). Evaluation of treatment response of radiotherapy in head and neck cancer with Fluorine-18-Fluorodeoxyglucose. *J. Nucl. Med.*, **29**, 1521–1525.

MINN, H. & SOINI, I. (1989). [18F]Fluorodeoxyglucose scintigraphy in diagnosis and follow up of treatment in advanced breast cancer. *Eur. J. Nucl. Med.*, **15**, 61–66.

NICHOLSON, S., HALCROW, P., FARNDON, J.R., SAINSBURY, J.R., CHAMBERS, P. & HARRIS, A.L. (1989). Expression of epidermal growth factor receptors associated with lack of response to endocrine therapy in recurrent breast cancer. *Lancet*, **jan 28**, 182–184.

NÅGREN, K. (1992). Quality aspects in the preparation of $^{11}$C-methionine. In *PET Studies on Amino Acid Metabolism and Protein Synthesis*. Comar, D., Heiss, W.-D. & Mazoyer, B. Kluwer Academic Publishers: Dordrecht (in press).

OLDENDORF, H. (1974). Expression of tissue isotope distribution. *J. Nucl. Med.*, **15**, 725–726.

PATLAK, C.S., BLASBERG, R.G. & FENSTERMACHER, J.O. (1983). Graphical evaluation of blood-to-brain transfer constants from multiple-time uptake data. *J. Cereb. Blood Flow Metab.*, **3**, 1–7.

SPINKS, T.J., JONES, T., GILARDI, M.C. & HEATHER, J.D. (1988). Physical performance of the latest generation of commercial positron scanner. *IEEE Trans. Nucl. Sci.*, **35**, 721–725.

STRAUSS, L.G. & CONTI, P.S. (1991). The applications of PET in clinical oncology. *J. Nucl. Med.*, **32**, 623–648.

WAHL, R.L., CODY, R., ZASADNY, K., HUTCHINS, G. & HELVIE, M. (1991). Active breast cancer chemohormonotherapy sequentially assessed by FDG PET: early metabolic decrements precede tumor shrinkage. *J. Nucl. Med.*, **32** (suppl), 982.

WOODARD, H.W., BIGLER, R.B., FREED, B. & RUSS, G. (1975). Expression of tissue isotope distribution. *J. Nucl. Med.*, **16**, 958–959.